# Automated ligand fitting by core-fragment fitting and extension into density

**DOI:** 10.1107/S0907444906017161

**Published:** 2006-07-18

**Authors:** Thomas C. Terwilliger, Herbert Klei, Paul D. Adams, Nigel W. Moriarty, Judith D. Cohn

**Affiliations:** a Los Alamos National Laboratory, Mailstop M888, Los Alamos, NM 87545, USA; b Bristol-Myers Squibb Pharmaceutical Research Institute, PO Box 4000, Princeton, New Jersey 08543-4000, USA; c Lawrence Berkeley National Laboratory, One Cyclotron Road, BLDG 64R0121, Berkeley, CA 94720, USA

**Keywords:** model building, model completion, shape analysis

## Abstract

A procedure for fitting of ligands to electron-density maps by first fitting a core fragment of the ligand to density and then extending the remainder of the ligand into density is presented. The approach was tested by fitting 9327 ligands over a wide range of resolutions (most are in the range 0.8–­4.8 Å) from the Protein Data Bank (PDB) into (*F*_o_ − *F*_c_)exp(*i*ϕ_*c*_) difference density calculated using entries from the PDB without these ligands. The procedure was able to place 58% of these 9327 ligands within 2 Å (r.m.s.d.) of the coordinates of the atoms in the original PDB entry for that ligand. The success of the fitting procedure was relatively insensitive to the size of the ligand in the range 10–100 non-H atoms and was only moderately sensitive to resolution, with the percentage of ligands placed near the coordinates of the original PDB entry for fits in the range 58–73% over all resolution ranges tested.

## Abstract 2

An automated ligand-fitting procedure has been developed and tested on 9327 ligands and (*F*_o_ − *F*_c_)exp(*i*ϕ_*c*_) difference density from macromolecular structures in the Protein Data Bank.

## 1. Introduction

Fitting of ligand density is an important step in the completion of macromolecular structures. It is often carried out as one of the very last steps in structure determination, after essentially the entire macromolecule and most solvent molecules have been fitted and refined. In pharmaceutical settings, many hundreds of structures may be solved in which the principal difference between these structures is the ligand (*e.g.* Tickle *et al.*, 2004 ▶).

The ligand-fitting step has often been carried out using interactive graphics tools (Jones *et al.*, 1991 ▶), but more recently several techniques have been developed that automate this process (Diller *et al.*, 1999 ▶; Oldfield, 2001 ▶; Tickle *et al.*, 2004 ▶; Zwart *et al.*, 2004 ▶; Evrard *et al.*, 2006 ▶; Emsley & Cowtan, 2004 ▶). The *X-Ligand* (Oldfield, 2001 ▶), *Coot* (Emsley & Cowtan, 2004 ▶) and *BLOB* (Diller *et al.*, 1999 ▶) methods identify density that fits a predefined conformation of the ligand and then adjust the conformation of the ligand to optimize this fit. In contrast, the *ARP*/*wARP* method identifies atomic features of a large compact region of high density in a map and interprets them in terms of the connectivity of the ligand (Zwart *et al.*, 2004 ▶; Evrard *et al.*, 2006 ▶). A third method has recently been described in which the shape of the density to be fitted is described with a spine-tracing algorithm that is relatively insensitive to noise which is then used as a template for fitting the ligand (Aishima *et al.*, 2005 ▶). Each of these methods can work very well, particularly for ligands of small or moderate size (up to about 50 non-H atoms) at moderate to high resolution (<2.5 Å). Fitting larger ligands with many rotatable bonds and fitting at lower resolution remains somewhat more difficult.

We have developed an approach to ligand fitting that is tailored to the fitting of large ligands and that can be used at both high and lower resolution. The basic idea is very simple: it is to find the location of any rigid part of the ligand (the core) and then to build the remainder of the ligand from this core by following the density, keeping in mind the stereochemical constraints of the ligand. This approach is attractive because it is similar to the approach that an experienced crystallographer would use. More importantly, it is suitable for large flexible ligands because the process is sequential and scales relatively linearly with the size of the ligand. We describe here the method and its application to over 9000 ligands from the PDB.

## 2. Methods

### 2.1. Geometrical analysis of a ligand

We assume that the conformation of the ligand in the structure to be modeled can be generated from another conformation of the same ligand by simple rotations around bonds. A few simple rules are used to decompose the ligand into a set of overlapping fragments, each of which has no internal rotatable bonds but which is connected to at least one other fragment through a rotatable bond. Only non-H atoms are considered in the analysis of a ligand. These rules are, owing to their simplicity, quite incomplete; they are intended to give a first approximation to the geometrical features of the ligand.

Our rules are as follows.

(i) Any two atoms separated by less than the sum of their ‘maximum half-bond lengths’ (defined below) are bonded.
(ii) Any set of atoms in a ring or set of rings with 20 or fewer atoms are in a fixed arrangement.
(iii) Any two atoms *A* and *C* bonded to a central atom *B* are in a fixed arrangement (*i.e.* the angle *A*—*B*—*C* is fixed).
(iv) Any set of four bonded atoms *A*—*B*—*C*—*D* that are coplanar are in a fixed arrangement (*i.e.* no rotations are allowed around the *B*—*C* bond).
(v) All sets of four bonded atoms *A*—*B*—*C*—*D* that are not specified as having a fixed arrangement can have any rotation about the *B*—*C* bond that does not place *A*—*B*—*C*—*D* within a specified tolerance (typically 0.1 Å) of being coplanar and that does not place any atoms separated by two or more bonds closer than a specified tolerance.

In this analysis, a group of atoms that is always in a fixed arrangement is considered a fixed ‘fragment’ of the ligand. Pairs of fragments that are connected (through a rotatable bond) will always have the two atoms that form this bond in common and will share no other atoms.

Once a ligand has been broken down into a set of rigid fragments connected by rotatable bonds using these rules, it is simple to construct possible conformations of the ligand. Firstly, the location and orientation of any rigid fragment is fixed. Any one of the fragments that are connected to this fixed fragment is then placed. The placement of this second fragment is determined by the bond that connects the two fragments and by the rotations allowed around this bond by rule (v) above. This process is repeated until all fragments are placed. The procedure can start with any of the rigid fragments and any order of addition of fragments connected to each other can be followed.

A limited default set of maximum half-bond lengths is used to identify commonly bonded atoms. These are essentially the half-bond lengths for these atoms plus a tolerance of about 0.1–0.3 Å. The maximum half-bond-lengths used are C, N, O, 0.8 Å; S, Br, I, 1.5 Å; P, F, 1.0 Å. The algorithm is not very sensitive to these values because the van der Waals radii of most atoms are far greater than their half-bond lengths so that there is usually little question as to whether two atoms are bonded. Maximum half-bond lengths for atoms that are not in the default set are estimated from the distance to the nearest other atom in the ligand and the half-bond length of that atom.

Our rules have limitations, but they are sufficient in many cases to identify most or all of the rotatable bonds. A general limitation of the approach is that the bond angles and lengths are all assumed to be identical in the ligand to be fitted and the ligand used to generate it. While significant, this limitation can in principle be overcome by subsequent refinement of the ligand structure. Another significant limitation is that the conformations of all the atoms in a ring are assumed to be fixed. While nearly true for aromatic rings, rings such as those in sugars can have alternative conformations that are quite different from each other. Similarly, two groups of atoms connected by an *sp* ^2^-hybridized bond are treated as fixed, while they could have either of two possible configurations. These limitations mean that a complete search for the conformation of a ligand that has more than one possible conformation of atoms in a ring or about an *sp* ^2^-bonded pair of atoms needs to be carried out more than once, beginning with examples of the ligand that have each of these conformations. In essence, the same ligand with different conformations of these types needs to be treated as two different ligands in our approach. We use antibumping constraints to ensure that atoms in the ligand avoid serious overlap with other atoms in the ligand and with other atoms in the structure of the macromolecule. If any two atoms in the ligand are not bonded to each other, they must be separated by at least the sum of their maximum half-bond lengths plus 1 Å or by the distance that they are separated by in the starting ligand conformation minus 0.5 Å, whichever is smaller.

### 2.2. FFT-based identification of the location and orientation of a core fragment of a ligand

Our approach to modeling a ligand begins with finding plausible placements of core fragments of the ligand in density. We use a simple approach to limit the search for the locations of these fragments to a small region within the unit cell by only searching within and near the largest and highest contiguous region of density in the map. This approach is similar to that used in several other ligand-fitting algorithms (Oldfield, 2001 ▶; Zwart *et al.*, 2004 ▶; Emsley & Cowtan, 2004 ▶). The identification of the location of this region is carried out in two steps. Firstly, a threshold of density is chosen such that the volume of the largest contiguous region within the map where all grid points are at or above this threshold is approximately the same as the volume of the ligand. The ligand is then assumed to be within this region. To speed up subsequent FFT-based convolution searches, the electron density within a box approximately 20 Å on a side, centered on this region of contiguous high density, is used to create a small pseudo-map in space group *P*1 with the same grid spacing as the original map and all the searches are performed within this small map.

The positions and orientations of core fragments of the ligand are identified using an FFT-based convolution search (*e.g.* Cowtan, 1998 ▶; Terwilliger, 2001 ▶). Each core fragment is placed at the origin of the small cell of electron density in space group *P*1 created above and possible orientations are then constructed (typically at 40° intervals) and used in a convolution search for similarly shaped density in the map. The orientation and positions (typically 300) yielding the highest overlap between the core fragment and the map are then refined based on correlation coefficient of density calculated from the core fragment and density in the map and the top refined placements (typically 100) are saved. These top placements of each of several rigid fragments of the ligand form the starting points for ligand-building trials.

### 2.3. Building a ligand by iterative extension into density

Once a rigid core fragment has been placed at a particular location and in a particular orientation in the unit cell, building the remainder of the ligand consists of an iterative procedure. A fragment that can be connected to the already built part of the ligand but which is not yet placed is picked, a placement for this fragment is chosen and the new partial or complete ligand is scored as described below. Typically, possible orientations for the additional fragment are sampled at intervals of 20°. At any point, a list of top-scoring partial and complete ligands is maintained (typically 300). The scoring procedure is designed to favor larger (*i.e.* more complete) ligands as long as the density is positive. The procedure terminates when all the top-scoring ligands have served as templates for addition of further fragments and no new top-scoring ligands are found.

### 2.4. Scoring of a fit of a partially built ligand to density

In this ligand-fitting procedure, the central criteria for choosing a particular configuration of a ligand or partially built ligand is the fit of the ligand to the density. Aside from the initial fitting of core fragments to density described above, we use a simple score for this fit that is based on the density at the coordinates of the *N* atoms in the ligand (ρ_*i*_) and on the atomic number of each atom in the ligand (*Z_i_*). The score *Q* is given by
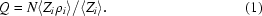
This score has the desirable property that it generally increases with an increase the number of atoms placed, increasing the density at coordinates of atoms and increasing the correlation of density with atomic number.

An additional criterion is used to help ensure that all atoms in the ligand are above a threshold of minimum density. Typically, the minimum allowed density for any atom in the ligand is −1.0 times the r.m.s. of the map.

Configurations of partially built cyclic ligands that cannot possibly be made to cyclize are also eliminated. These are identified as configurations in which any two fragments are so far apart that no arrangement of the fragments linking them can possibly connect.

### 2.5. Recombination among separately built copies of a ligand and ligand completion

The procedure described above produces a list of partial and complete fitted ligands ranked by their fit to the electron-density map. These fitted ligands will have been generated starting from different core fragments and therefore will have in general been traced beginning from different parts of the density for the ligand. We developed a procedure for recombination among the fitted ligands to increase the quality of the fit to density followed by further addition of fragments to increase the completeness of the ligand. In this step the requirement that all atoms be above a minimum threshold is removed so as to create essentially fully complete ligands even if some atoms do not match the density. The recombination is carried out among pairs of ligands in the top group of saved ligands (typically 100). A new ligand is built from fragments of the two existing ligands, beginning with a fragment from one ligand, adding fragments connected to the original fragment one at a time, then at selected points crossing over to the corresponding fragment from the other ligand. Crossovers of this kind are made only between two copies of a fragment where the coordinates of atoms in the two copies match within a specified tolerance (typically 1 Å). During the creation of crossovers, the score of a ligand is modified based on the r.m.s.d. between atoms in the fragments, with the offset in score *O* for each crossover given typically by
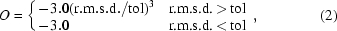
where tol is the tolerance above (typically 1 Å).

## 3. Results and discussion

### 3.1. Fitting ligands from the PDB

We tested our algorithm for ligand fitting by using it to fit 9327 ligands from 6209 X-ray structures in the PDB (Berman *et al.*, 2000 ▶). The high-resolution limits of the corresponding PDB entries ranged from 0.8 to 10 Å, with all but two in the resolution range 0.8–4.8 Å. The PDB entries and ligands chosen were from X-ray structures in the November 2004 release of the PDB for which all of the following held: (i) the entry contained coordinates for at least one polypeptide macromolecule with a minimum of 20 amino acids, (ii) the entry contained coordinates for at least one non-macromolecule, defined by an mmCIF entity_id (Greer *et al.*, 2002 ▶), with 6–150 heavy atoms and, if a polypeptide, containing no more than two residues, (iii) the entry had structure-factor amplitudes or intensities that, with minor automatic editing, could be read using the *CCP*4 program *cif2mtz* (Collaborative Computational Project, Number 4, 1994 ▶), (iv) at least one ligand in the entry could be analyzed by our procedure as described in §[Sec sec2]2 and (v) if the entry was one of a series of sequentially named PDB entries all containing the same ligand, the entry was the first in that series. Criterion (v) was a simple way to remove entries that were near-duplicates of other entries. A total of 7025 PDB entries met criteria (i), (ii) and (iii). Of the 23 514 ligands in those entries, 952 (4%) were eliminated by criterion (iv). Furthermore, of the remaining 6881 PDB entries, 672 were rejected based on criterion (v), leaving 6209 unique PDB entries containing at least one polypeptide macromolecule and at least one ligand with associated structure-factor information.

For each PDB entry, one copy of each unique ligand was selected. A unique ligand was defined as an mmCIF entity containing a unique ordered list of hetero codes and non-H atom names. In this way, a set of 9327 ligands from 6209 PDB entries was chosen to represent nearly all of the unique ligand–PDB entry combinations with associated structure factors available in the November 2004 release of the PDB. The 9327 ligands represent 3299 unique ligands.

The 9327 ligands were fitted in the following way. For each ligand, two separate PDB files were generated: one containing non-H-atom records for the ligand only (ligand file) and one containing all other non-H-atom records in the same PDB entry (minus file). These files were generated from an Oracle database that had been populated using v.1.5.1 of the *openMMS Toolkit* (Greer *et al.*, 2002 ▶) with data from mmCIF files obtained from ftp://beta.rcsb.org/pub/pdb/uniformity/data/mmCIF/divided. Structure factors were calculated using the coordinates with the ligand removed and an (*F*_o_ − *F*_c_)exp(*i*ϕ_*c*_) difference map was calculated. The correlation coefficient of the original ligand with this *F*_o_ − *F*_c_ difference density (cc_orig_) was noted and used as a basis for classifying the quality of the difference density. Ligand fitting was then carried out using a second ligand file generated from another copy of the same ligand. In most cases, the second ligand file was derived from a different PDB entry (6930 ligands). If this was not possible, the second ligand file was derived from a second instance of the ligand in the same PDB entry (862 ligands) or, if there were no other instances of the unique ligand (1535 ligands), the original ligand was used but oriented arbitrarily so that it could not be simply replaced.

The difference map, the second copy of the ligand and a mask calculated from the coordinates without the ligand were used as the inputs to the ligand-building procedure described in §[Sec sec2]2. For each combination of map, ligand and mask, the number of fragments used in the FFT convolutions, the number of placements of each fragment considered and the number of top solutions saved were initially set to low values and increased over five fitting attempts until all attempts were made or a correlation of the ligand with density of at least 0.75 was obtained. This procedure was carried out in order to speed up the fitting of ligands that could be readily fitted while still fitting the more complex ligands.

Table 1 ▶ summarizes the results of fitting ligands into difference density from entries in the PDB after removing ligands one at a time. The *F*_o_ − *F*_c_ difference maps were classified according to the correlation of difference density and density calculated from the original ligand in the PDB entry (cc_orig_). There were 6590 ligand–PDB entry combinations with clear density for the ligand as found in the PDB entries (cc_orig_ ≥ 0.75). For these cases, the mean correlation after fitting was lower (0.76) than the mean correlation for the original ligands (0.85). However, 41% of the fitted ligands had r.m.s. coordinate differences relative to the original ligands of less than 1.0 Å and 71% had an r.m.s.d. of 2.0 Å or less from the coordinates of the original ligands. Only 310 (5%) were placed very differently from the original ligands (with an r.m.s.d. of more than 10 Å from the coordinates of the original ligands). For the 2737 ligand–PDB entry combinations with weak density for the ligand as found in the PDB entries (cc_orig_ < 0.75), 41% had an r.m.s.d. of more than 10 Å from of the coordinates of the original ligands; however, the mean correlation of the fitted ligands (0.60) was nearly the same on average as that of the original ligands as found in the PDB (0.61), suggesting that equally well fitting density was found in most cases.

For the entire set of 9327 ligand–PDB entry combinations, 5421 (58%) were rebuilt with an r.m.s.d. of 2.0 Å or less from of the coordinates of the original ligands and 1425 (15%) had an r.m.s.d. more than 10 Å from of the coordinates of the original ligands.

In an actual fitting experiment, water molecules would normally either already be placed (including placements in the ligand-binding site) or would never be placed at all. Furthermore, the structure would normally be refined without the ligand present, leading potentially to weaker density for the ligand. We did not test the effect of refinement, but we did test the effect of including the solvent. We expected that solvent molecules in the PDB entries could have an effect on the ligand-fitting procedure both through exclusion of some locations from being considered as a place for a ligand to be located and through contributions to structure factors. We tested this effect by carrying out a matched pair of fitting experiments which differed in that all water molecules were removed prior to map calculation and fitting for one experiment. In these experiments, ligand fitting was carried out beginning with the original ligand from the PDB entry under consideration. We tested an arbitrarily chosen set of 2641 ligand–protein pairs (including multiple instances of a ligand from the same PDB entry) fitted with and without solvent molecules and the two approaches were found to yield similar but not identical results. The mean of the correlation coefficient between matched ligand–PDB entry pairs was 0.02 higher with solvent. The fraction of ligands rebuilt with an r.m.s.d. of 2.0 Å or less from of the coordinates of the original ligands was 67% for this set of ligand–PDB entries including water molecules and 58% after removing them. These results suggest that the fitting obtained with the inclusion of water molecules as detailed in Table 1 ▶ may be slightly better than the results would be in an actual fitting experiment.

### 3.2. Sensitivity to starting ligand conformation

**Figure 1:**
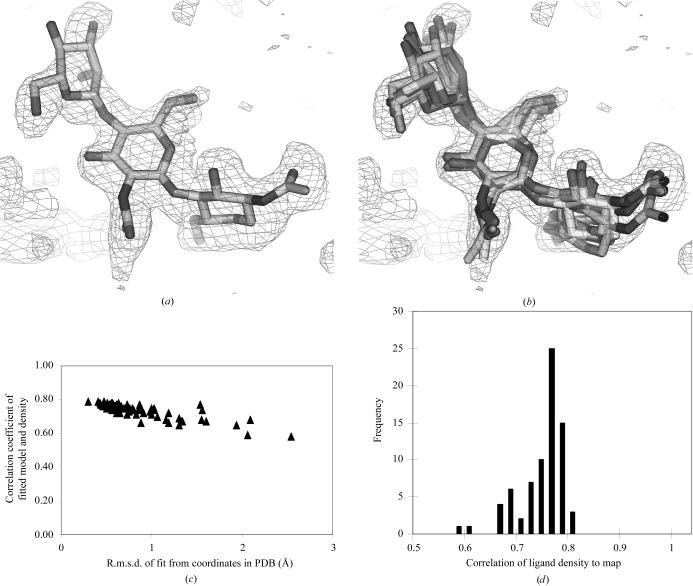
(*a*) *F*_o_ − *F*_c_ difference density for NAG-NAG-MAN (PDB entry 1d7d, 1.95 Å; Hallberg *et al.*, 2000 ▶) fitted beginning with the same ligand from a different PDB entry. (*b*) The same map fitted beginning with ligands from five different PDB entries. (*c*) R.m.s.d. of fits beginning with NAG-NAG-MAN from 74 different PDB entries to the original ligand in PDB entry 1d7d and correlation coefficient of fitted ligand to the difference density map. (*d*) Histogram of r.m.s.d. of fits from (*c*).

We next examined the reproducibility of the procedure and sensitivity to the starting ligand conformation by fitting a NAG-NAG-NAM ligand 74 different times, each time using a different conformation of the ligand from an entry in the PDB. Fig. 1 ▶(*a*) illustrates the fit of a single copy of this ligand and Fig. 1 ▶(*b*) the fits of five copies. Fig. 1 ▶(*c*) shows the range of r.m.s.d. values of 74 fits from the coordinates in the original ligand in the PDB entry and the correlation coefficients of each fitted ligand to the *F*_o_ − *F*_c_ density map and Fig. 1 ▶(*d*) shows histograms of the correlation coefficients. Most of the 74 fits yield correlations between 0.7 and 0.8 and r.m.s.d. values less than 1.5 Å. This indicates that the procedure, while not giving precisely the same conformation in every trial beginning with a different conformation of the ligand, gives a relatively reproducible fit of the ligand to density.

### 3.3. Sensitivity to resolution and size of ligand

**Figure 2:**
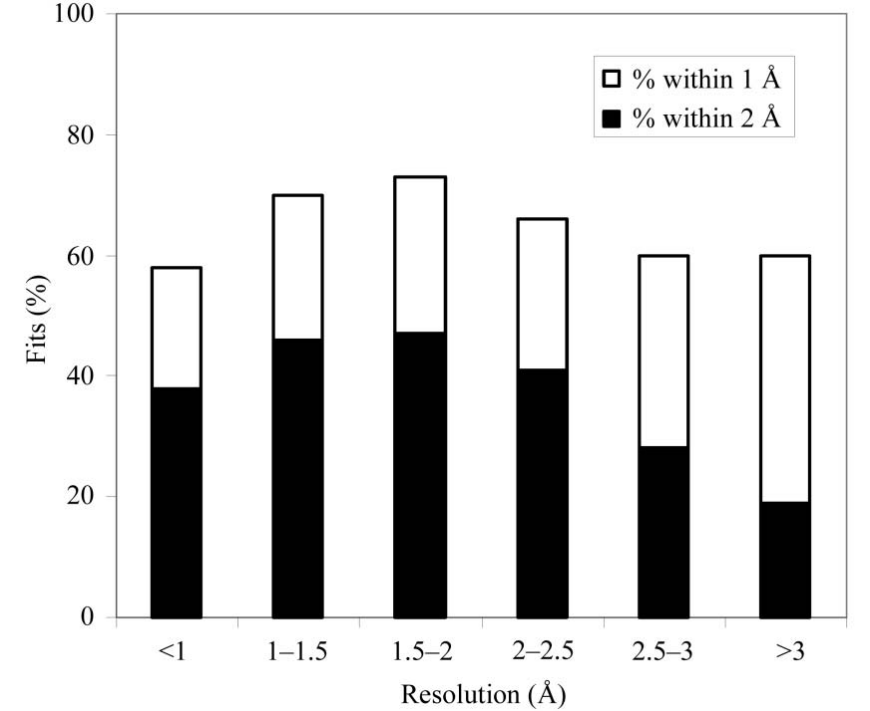
Histogram of number of fits with an r.m.s.d. to the original coordinates in the PDB within 1 Å (filled bars) and within 2 Å (entire length of bars including filled and unfilled parts) as a function of the resolution of the maps, considering only ligand–PDB combinations where the original ligand had a correlation with the *F*_o_ − *F*_c_ map of 0.75 or greater.

Fig. 2 ▶ summarizes the fits of ligands to *F*_o_ − *F*_c_ density from the PDB according to the resolution of the data used. In order to focus on the resolution of the data and minimize effects of differing qualities of maps at different resolutions, only the 6590 ligand–PDB combinations for which the correlation of the original ligand to the *F*_o_ − *F*_c_ map is at least 0.75 are included. Fig. 2 ▶ indicates that the ligand-fitting procedure is able to fit about 60% of ligands to within about 2 Å of the coordinates of the original ligand in the PDB, relatively independent of the resolution of the map, but highest in the resolution range 1.0–2.0 Å. The percentage of ligands that are fitted very accurately (within an r.m.s. of 1 Å of the coordinates of the original ligand in the PDB) in contrast is much higher for ligands fitted in the range 1.0–2.0 Å than for ligands fitted at resolutions >3.0 Å.

**Figure 3:**
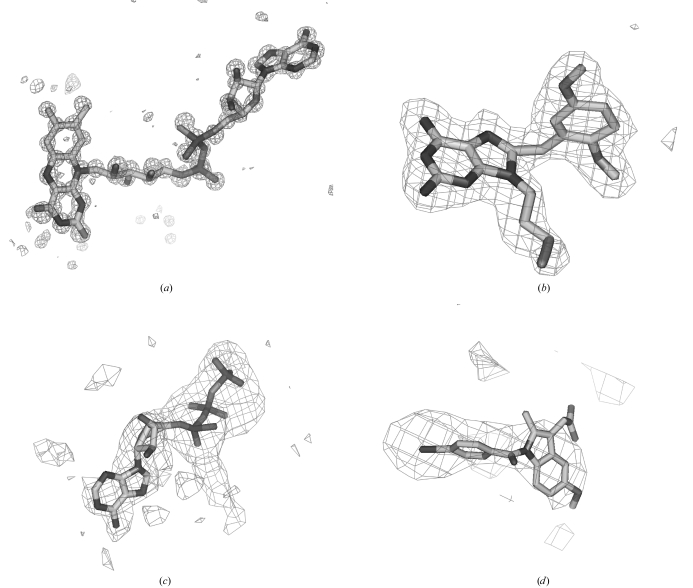
Fitting ligands at resolutions from 0.95 to 4.5 Å. (*a*) Fit of *F*_o_ − *F*_c_ difference density at 0.95 Å of FAD (PDB entry 1n1p; Lario & Vrielink, 2003 ▶). (*b*) Fit at 2.2 Å of 8-(2,5-dimethoxybenzyl)-2-fluoro-9-pent-9*H*-purin-6-ylamine (PDB entry 1uyi; Wright *et al.*, 2004 ▶). (*c*) Fit at 3 Å of ATP (PDB entry 1nbm; Orriss *et al.*, 1998 ▶). (*d*) Fit at 4.5 Å of 1-(4-iodobenzoyl)-5-methoxy-2-methyl-indole-3-acetic acid (PDB entry 1pgf; Loll *et al.*, 1996 ▶).

Fig. 3 ▶ shows examples of fitting ligands at resolutions from 0.95 to 4.5 Å. The ligand-fitting procedure places the ligands in positions and conformations at each resolution that are compatible with the *F*_o_ − *F*_c_ difference maps, though the precision with which the resulting model can be defined is clearly much better for the higher resolution maps than those at lower resolution.

**Figure 4:**
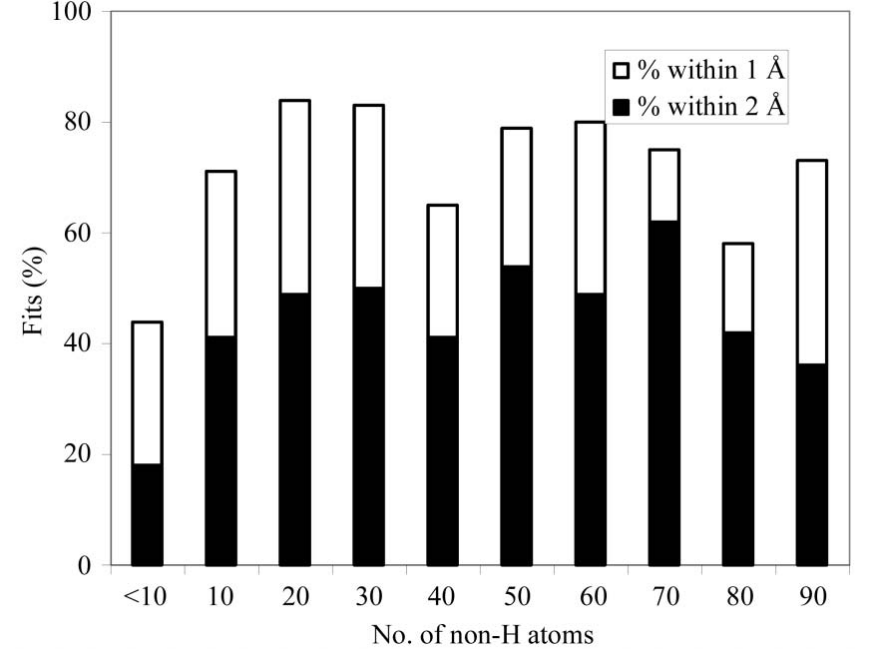
Histogram of number of fits with an r.m.s.d. to the original coordinates in the PDB within within 1 Å (filled bars) and within 2 Å (entire length of bars including filled and unfilled parts) as a function of the number of non-H atoms in the ligand, considering only ligand–PDB combinations where the original ligand had a correlation with the *F*_o_ − *F*_c_ map of 0.75 or greater. The number of non-H atoms in the ligands considered ranged from 6 to 150.

Fig. 4 ▶ summarizes the fits of ligands as a function of the number of non-H atoms in the ligand, limiting the ligand–PDB combinations to the 6590 for which the correlation of the original ligand to the *F*_o_ − *F*_c_ map is at least 0.75. It indicates that very small ligands are fitted relatively poorly; just 44% are fitted within an r.m.s.d. of 2 Å of the original ligand in the PDB. In contrast, the percentages of ligands with 10–90 atoms fitting within an r.m.s.d. of 2 Å of the original ligand in the PDB is high and relatively constant, with a mean value of 74 ± 8%.

## 4. Conclusion

We have developed a procedure for fitting flexible ligands that is useful over a wide range of resolutions and that works well for ligands with over 90 non-H atoms. There remain significant improvements that could be made to the procedure. In particular, our simplistic method for analysis of allowable torsion angles and for ligand geometry could be replaced with results from semi-empirical or quantum-mechanical calculations. This would remove the limitations on the method arising from assuming that atoms in rings have fixed relative positions, for example. Other significant improvements that could be made might include a more detailed analysis of alternative choices for the location of the ligand, optimization of the scoring function used and optimization of the choices of the numbers of partially built ligands to keep at each stage. Additionally, subsequent to building of ligand models, the refinement of these models would be expected to improve their geometries and fit to the density. We expect that this procedure may prove useful as one of the tools that can be routinely applied during the final stages of model building for macromolecular crystallography to assist in model completion. Additionally, non-bonded interactions among atoms in the ligand may be useful in optimizing the conformation of the ligand.

## Figures and Tables

**Table 1:** Fitting *F*_o_ − *F*_c_ density from PDB entries after removing ligands

*F*_o_ − *F*_c_ density correlation with original ligand from PDB entry	cc_orig_ ≥ 0.75	cc_orig_ < 0.75	All
No. of ligand–entry combinations	6590	2737	9327
Mean ligand-density correlation with original ligand from PDB entry	0.85	0.61	0.78
Mean fitted ligand-density correlation	0.76	0.60	0.72
R.m.s.d. ≤ 1.0 Å	2715 (41%)	289 (11%)	3004 (32%)
R.m.s.d ≤ 2.0 Å	4666 (71%)	755 (28%)	5421 (58%)
R.m.s.d. > 10 Å	310 (5%)	1115 (41%)	1425 (15%)
